# Current status of medical research among undergraduate medical students in China: a nationwide questionnaire survey

**DOI:** 10.3389/fmed.2025.1593233

**Published:** 2025-07-10

**Authors:** Liuyan Xu, Jian Zhou, Chengxi Fan, Kailin Huang, Abudukerimu Muyesha, Rurong Wang, Xuehan Li

**Affiliations:** Department of Anesthesiology, West China Hospital of Sichuan University, Chengdu, Sichuan, China

**Keywords:** medical research, medical undergraduates, undergraduate medical research, medical education, questionnaire study

## Abstract

The increasing demand for physician-scientists highlights the essential role of medical schools in fostering the development of these professionals. This study aims to assess the current status of research engagement, attitudes toward research, and barriers to participation among Chinese undergraduate medical students. A cross-sectional, nationwide questionnaire survey was conducted in February 2024 using an online platform, with responses from 3,423 students (effective rate: 87.79%). Furthermore, we have designed a questionnaire among physician-scientists with experience in teaching undergraduates about research at West China Hospital of Sichuan University, resulting in 51 responses being collected. The questionnaires were developed based on existing literature and expert input and underwent pilot testing. Reliability and validity were confirmed via Cronbach’s alpha and KMO-Bartlett tests. Statistical analyses included chi-square, ANOVA, and multivariate regression. Results showed that 70.6% of students had engaged in research at least once, but reported limited exposure and a lack of knowledge as major barriers. Males and preventive and basic medicine students demonstrated higher research interest and participation. Factors such as gender, major, academic year, and prior research involvement significantly influenced students’ attitudes and perceptions of scientific research. The faculty considered the presence of undergraduate research engagements to be a significant assessment criterion. They opined that the prevailing undergraduate research climate was inadequate. The findings underscore the need for structured, competency-based research training and institutional support.

## 1 Introduction

Physician-scientists are essential to basic research, clinical translation, and biotechnology advancements, all of which drive the growth of the life sciences industry in any thriving health economy (1). Their unique dual expertise in medicine and research enables the identification of practical solutions for complex scientific challenges. At the same time, clinically focused physicians require strong scientific literacy to provide evidence-based care, particularly when confronting emerging health threats such as COVID-19, based on the latest research findings (2).

Globally, there is a well-documented decline in the number of physician-scientists, accompanied by an aging workforce, raising concerns about the sustainability of this crucial group (3). In China, although national-level data on the shortage of physician-scientists are limited, some localized studies suggest similar trends, highlighting the urgency of cultivating research-capable clinicians (4, 5). However, comprehensive nationwide assessments of undergraduate medical students’ involvement, attitudes, and barriers toward medical research are still lacking.

Medical schools play a critical role in addressing this gap by offering foundational research courses and providing real-world research opportunities, such as exposure to the scientific publication process. These experiences significantly contribute to the development of future physician-scientists. The decision of a clinician to pursue a research career and nurture their research interests is profoundly influenced by the experiences gained during medical school. Literature suggests that medical student involvement in research helps cultivate valuable skills such as communication, teamwork, and time management, as well as boosting motivation for a research career. Additionally, this involvement fosters critical thinking, enhances presentation skills, and strengthens positive attitudes toward science and scientific methodology (6, 7). Early engagement in research also has long-term effects on physicians’ scientific careers, with studies indicating that medical students who engage in writing during their studies are not only more prolific in publishing, but also achieve higher citation impacts and are more likely to continue publishing after graduation (8).

In our study design, we differentiated “attitudes” (students’ feelings and values toward research) “perceptions” (their understanding of the relevance and process of research), and “research engagement” (actual participation in research activities), to comprehensively evaluate factors influencing undergraduate research involvement (9). The term “barriers” in this study refers to multiple dimensions, including institutional factors (such as limited mentorship and research infrastructure), personal factors (including lack of time and insufficient skills), and societal factors (such as cultural attitudes toward research careers) that hinder student participation in research activities (6).

Based on this background, the current study aims to provide an in-depth understanding of the status of medical research engagement among undergraduate medical students in China. First, we assess the actual participation of students in medical research. Second, we explore their attitudes toward and perceptions of research. Additionally, we analyze the main barriers that hinder their research involvement. Finally, we propose practical recommendations to promote the development of research training within Chinese undergraduate medical education, ultimately supporting the cultivation of more research-competent clinicians. This nationwide questionnaire survey offers valuable evidence for policymakers and educators to design targeted interventions that enhance medical students’ research literacy, fostering a stronger integration of clinical practice and scientific inquiry in China’s healthcare system.

## 2 Materials and methods

### 2.1 Study design and sampling

This cross-sectional study was conducted in February 2024 among undergraduate medical students across China. Data collection was administered through an online platform (Wenjuanxing, a widely-used Chinese survey tool),^1^ ensuring voluntary and anonymous participation. Inclusion criteria included being enrolled as a full-time undergraduate student in a recognized Chinese medical school. Exclusion criteria encompassed incomplete responses, duplicated entries (verified by timestamp and IP pattern), and inconsistent demographic data. A convenience sampling method was used; however, care was taken to invite students from diverse geographic regions and institutional types to maximize representativeness. Duplicate entries were screened via IP address and timestamp analysis. Shared computers/NAT networks were flagged for manual review (e.g., checking response patterns and demographic consistency).

### 2.2 Questionnaire development and validation

The questionnaire was developed after reviewing validated tools in the literature (10–13) and refined with input from five medical education experts. It consisted of 23 items divided into three sections: (1) demographic and educational background, (2) research engagement and experiences, and (3) attitudes, barriers, and career aspirations.

Prior to full deployment, a pilot test was conducted among 50 medical students at West China Hospital. Based on feedback, several items were reworded for clarity. Reliability was assessed using Cronbach’s alpha (*α* = 0.89 for the undergraduate instrument; α = 0.83 for the faculty instrument), indicating good internal consistency. Validity was evaluated through the Kaiser-Meyer-Olkin (KMO) measure and Bartlett’s test of sphericity. The KMO values were 0.97 and 0.71 respectively, both significant at *p < 0*.001.

### 2.3 Study instruments

The questionnaire consisted of 23 items and can be divided into three sections (the full questionnaire can be found in Supplementary material): Section 1 gathered gender, year of study, major, and self-rated research ability (scored 1–5 with descriptors). Section 2 explored previous research activities such as group participation, publications, patent applications, and motivations. Section 3 used a 5-point Likert scale to assess attitudes toward research, perceived institutional support, integrity education, and barriers.

Additionally, we developed a separate questionnaire aimed at exploring the perspectives of physician-scientists with experience in teaching undergraduates about research at the West China Hospital of Sichuan University. A total of 51 responses were collected.

### 2.4 Statistical analysis

Quantitative data were analyzed using SPSS version 26.0 (IBM Corp, Chicago, IL). Normality was assessed via the Kolmogorov–Smirnov test. Descriptive statistics were calculated for demographic data. Chi-square and Fisher’s exact tests compared categorical variables. For continuous data, independent t-tests or ANOVA were used if normally distributed; Mann–Whitney U or Kruskal–Wallis tests were applied otherwise. To identify predictors of research engagement, a multivariate logistic regression model was employed, adjusting for gender, major, academic year, and prior research exposure. Statistical significance was set at *p <* 0.05. Sensitivity analyses were conducted to assess robustness, including exclusion of outliers. For subgroup comparisons, Tukey’s HSD post-hoc test was used to adjust for multiplicity. Other analyses reported uncorrected *p*-values with interpretation caution due to exploratory aims.

## 3 Results

### 3.1 General characteristic of the population

**Figure 1:**
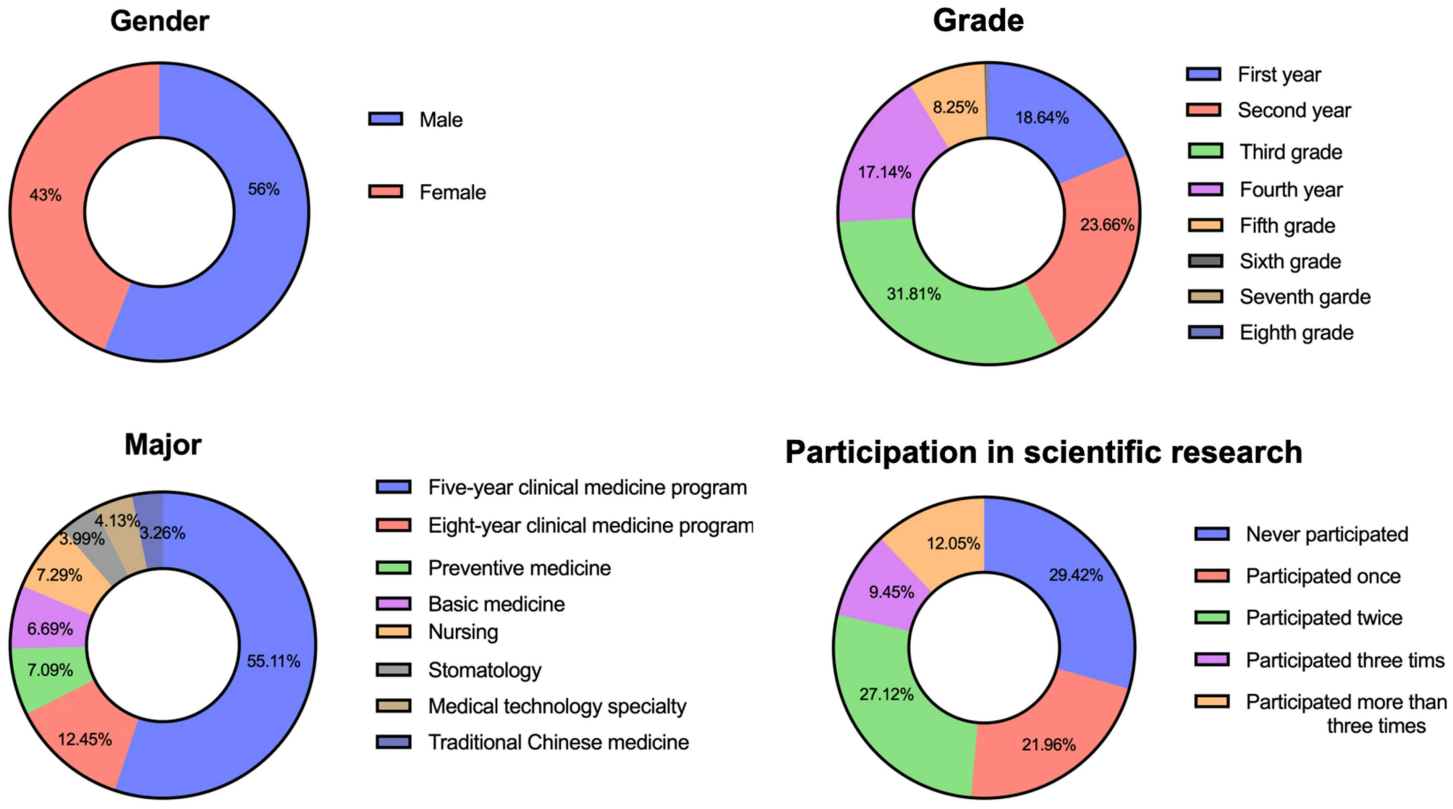
Medical students’ demographics and academic characteristics.

A total of 3,423 medical undergraduates from 39 medical schools participated in this study, with an effective response rate of 87.79% (3,005 out of 3,423). Among the participants, 1,684 (56%) were male and 1,321 (44%) were female. Third-year students constituted the majority (956, 31.8%), followed by second-year students (711, 23.7%), first-year students (560, 18.6%), fourth-year students (515, 17.1%), and fifth-year students (248, 8.3%). The survey included various medical majors, with the following distribution: clinical medicine (five-year program: 1,656, 55.1%; eight-year program: 374, 12.4%), preventive medicine (213, 7.1%), basic medicine (201, 6.7%), nursing (219, 7.3%), stomatology (120, 4.0%), medical technology (124, 4.1%), and traditional Chinese medicine (98, 3.3%). The demographic and academic summary information for the medical students is presented in Tables 1, 2 and Figure 1.

**Table 1:** Medical students’ demographics characteristics.

Students’ characteristics	Total *N* = 3,005	
	No.	%
Gender		
Male	1,684	56
Female	1,321	44
Major		
Five-year clinical medicine program	1,656	55.1
Eight-year clinical medicine program	374	12.4
Preventive medicine	213	7.1
Basic medicine	201	6.7
Nursing	219	7.3
Stomatology	120	4.0
Medical technology specialty	124	4.1
Traditional Chinese medicine	98	3.3
Grade		
First year	560	18.6
Second year	711	23.7
Third grade	956	31.8
Fourth year	515	17.1
Fifth grade	248	8.3
Sixth grade	8	0.3
Seventh grade	2	0.1
Eighth grade	5	0.2

**Table 2:** Medical students’ academic characteristics.

Students’ academic characteristics	No.	%
Participation in scientific research		
Never participated	884	29.4
Participated once	660	22
Participated twice	815	27.1
Participated three times	284	9.5
Participated more than three times	362	12
Scientific research experience		
Joined a research group	1,238	41.1
Wrote a paper	1,180	39.3
Published a paper	935	31.1
Applied for a patent	609	20.3
Other	6	0.2
Plans for a future career		
Not pursuing a master’s degree	347	11.54
Professional master’s degree	1,408	46.86
Academic master’s degree	928	30.88
A master’s degree abroad	115	3.82
Undecided about the degree	207	6.88
Barrier		
A lack of knowledge of medical research	2,135	71.04
A lack of exposure and opportunities	2,121	70.58
A lack of allotted time	1,698	56.12
A lack of mentoring and guidance	1,535	51.80
Other	42	1.40

### 3.2 Scientific research ability

Undergraduate medical students self-assessed their scientific research ability before enrolment and now (scores 1–5). A comprehensive assessment of research proficiency was conducted, with the average score for total research ability being 3.28 ± 1.23. Paired samples t-tests revealed statistically significant improvements (*p* = 0.044) in all research competencies when compared to pre-enrollment skills, indicating that current research abilities were superior to those assessed before enrollment.

**Figure 2:**
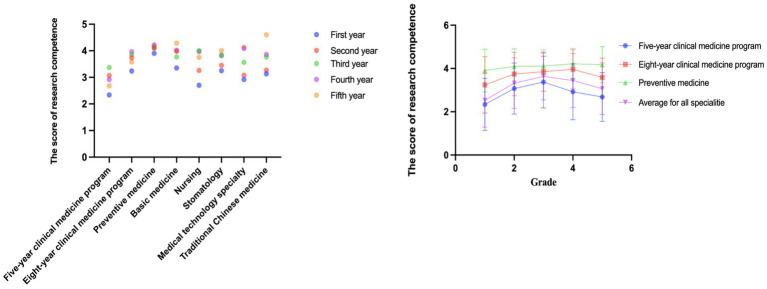
Scores of research competence in different majors and different grades.

Specialties in public health and preventive medicine scored the highest across all research competencies, likely due to their greater emphasis on courses in epidemiology and statistical analysis. ANOVA tests showed significant differences in the scores earned by students in different specialties. Third-year undergraduates scored the highest, followed by fourth-year students. This could be attributed to the undergraduate training program in China, where medical students in their fourth and fifth years are required to complete internships, which may reduce the time available for research training, thereby impacting their research abilities. Furthermore, statistically significant differences in research ability were found across academic years, except for the sixth, seventh, and eighth years in the clinical eight-year program. Table 3 and Figure 2 provide a detailed overview of the scientific research abilities of the undergraduates.

**Table 3:** Scientific research ability of medical undergraduates.

	Mean ± SD	*p* value
Scientific research ability		
The ability to innovate	3.3 ± 1.203	
The ability to design a subject	3.17 ± 1.204	
Knowledge of clinical research	3.16 ± 1.284	
Knowledge of basic research	3.17 ± 1.275	
Knowledge of research methods and processes	3.25 ± 1.227	
Searching and screening of literature	3.38 ± 1.157	
The use of a literature manager	3.34 ± 1.192	
The ability of data organization and statistical analysis	3.29 ± 1.221	
The ability of writing a medical paper	3.20 ± 1.255	
The overall research ability	3.28 ± 1.238	
Stratified by students’ characteristics		
Major^*^		**<0.001**
Five-year clinical medicine program	2.91 ± 1.262	
Eight-year clinical medicine program	3.79 ± 0.966	
Preventive medicine	**4.13 ± 0.770**	
Basic medicine	3.86 ± 0.935	
Nursing	3.40 ± 1.155	
Stomatology	3.65 ± 1.026	
Medical technology specialty	3.31 ± 3.62	
Traditional Chinese medicine	3.62 ± 1.214	
Grade^*^		**<0.001**
First year	2.53 ± 1.246	
Second year	3.32 ± 1.170	
Third grade	**3.63 ± 1.084**	
Fourth year	3.44 ± 1.250	
Fifth grade	3.06 ± 1.204	
Sixth grade	4.25 ± 0.707	
Seventh grade	2.50 ± 0.707	
Eighth grade	3.60 ± 1.673	
Gender^*^		**<0.001**
Male	**3.46 ± 1.171**	
Female	3.04 ± 1.280	

^*^One-way ANOVA.

### 3.3 Involvement in research activities

Overall, 70.6% of respondents reported having participated in at least one research-related activity. Participation was defined as involvement in original research projects, research groups, paper writing, patent application, or competition-based innovation activities. The most common activities included joining a research group (41.1%), writing a paper (39.3%), and publishing a paper (31.1%). Notably, 20.3% of students reported applying for a patent, which primarily referred to course-based innovation or student entrepreneurship projects, often incentivized by institutional programs. Students enrolled in the eight-year clinical program demonstrated the highest frequency and duration of participation. Comparative subgroup analysis indicated significantly higher participation among male students (*p* < 0.001), students majoring in preventive and basic medicine (*p* < 0.001), and third-year students (*p* < 0.001). Our findings also show that students who engaged in more research activities had higher ratings of research competence, indicating a positive correlation (Spearman’s rho = 0.104, *p* < 0.001) between research involvement and research skills. In the multivariate logistic regression model, male gender (OR = 1.52, 95% CI: 1.28–1.81), enrollment in preventive medicine (OR = 2.01, 95% CI: 1.45–2.78) or basic medicine (OR = 1.87, 95% CI: 1.32–2.65), and prior research involvement (OR = 3.12, 95% CI: 2.67–3.64) were significant predictors of research participation (all *p* < 0.001).

The main motivation for medical students’ participation in research was interest in the subject, followed by goals such as improving their academic performance, preparing for comprehensive exams, and peer pressure. An analysis of undergraduate students who planned to pursue a PhD revealed that they tended to spend more time on research, with their primary motivation being preparation for graduate school, distinguishing them from students who did not intend to enroll in a PhD program.

**Figure 3:**
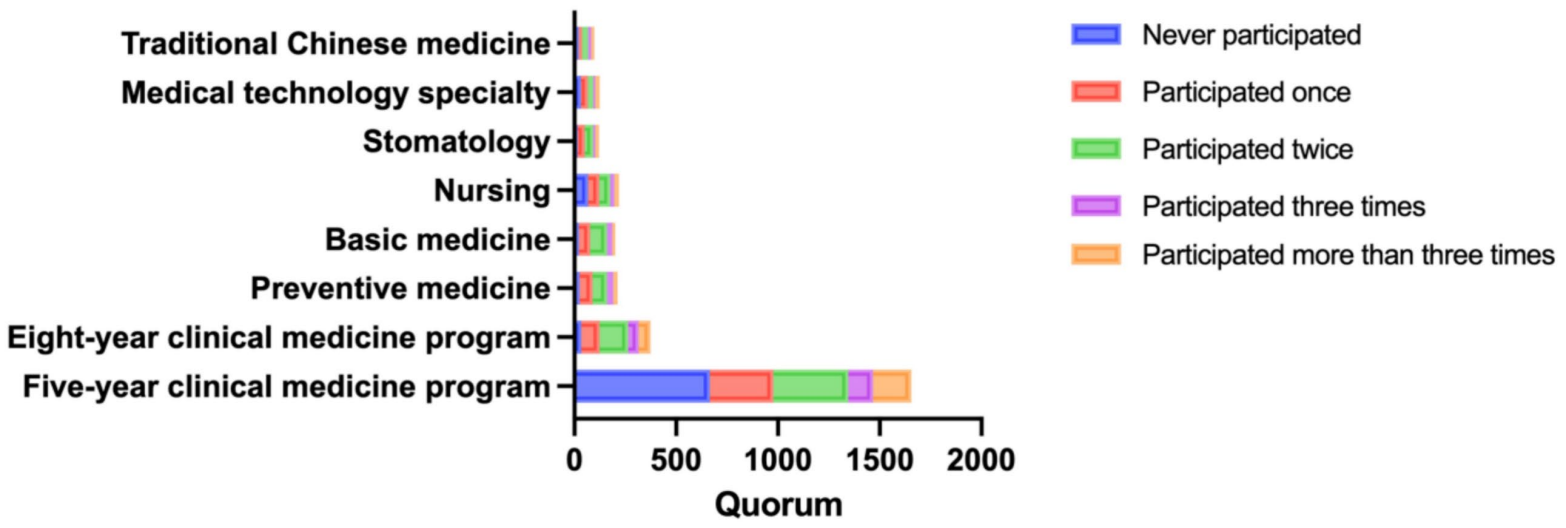
The participation in scientific research in different majors and different grades.

The questionnaire results indicated that the majority of students were first exposed to research during their second year. Furthermore, 85.5% of students had been exposed to research by their third year or earlier, with 34.8% of students expressing that research exposure should begin in the second year. Based on these findings, it is recommended that medical schools provide more research opportunities to younger undergraduates. Figure 3 illustrates participation in scientific research.

### 3.4 The attitudes toward medical research

Attitudes were assessed via a 5-point Likert scale. Students generally expressed positive views: 94.2% agreed that research is important to their future careers, and 90.5% were willing to participate in undergraduate research programs. Mean scores were significantly higher for students in basic medicine (mean = 24.5, SD = 3.78), followed by preventive medicine and the eight-year clinical program. Gender differences were statistically significant (*p* < 0.001), with male students reporting more favorable attitudes.

**Figure 4:**
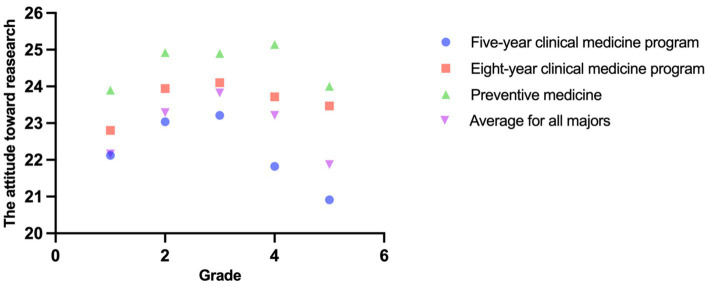
The attitudes toward medical research in different majors and different grades.

A positive correlation (Spearman’s rho = 0.41, *p* < 0.001) was found between a number of research activities and attitude score. Further multiple regression analysis (R^2^ = 0.252, *p* < 0.001), including predictors such as age, gender, and scientific research ability that previous research experience was the strongest predictor of positive attitudes (*β* = 0.36, *p* < 0.001). Other significant predictors included age and gender showed no significant association (both *p* > 0.05). The perspectives of undergraduate medical students on medical research are shown in Table 4 and Figure 4.

**Table 4:** The attitudes toward medical research.

		*p* value
The attitudes toward medical research		
Doing research is fun	3.87 ± 0.965	
Exposure to research opportunities is high	3.45 ± 1.008	
I would like to have more opportunities to participate in research activities	3.95 ± 0.897	
I think research is important in my future medical career	4.03 ± 0.881	
I am willing to participate in an undergraduate research teaching program	4.02 ± 0.884	
I think undergraduates should have a research teaching program	3.94 ± 0.888	
I think there are many of research activities organized by the university and related departments that students can participate in	3.88 ± 0.924	
I think research integrity is important	4.16 ± 0.878	
I have been taught about research integrity	3.89 ± 1.010	
Stratified by students’ characteristics		
Major^a^		**<0.001**
Five-year clinical medicine program	22.45 ± 4.40	
Eight-year clinical medicine program	23.83 ± 4.15	
Preventive medicine	24.43 ± 3.93	
Basic medicine	**24.54 ± 3.78**	
Nursing	23.16 ± 4.32	
Stomatology	23.57 ± 3.94	
Medical technology specialty	23.68 ± 4.24	
Traditional Chinese medicine	24.48 ± 4.55	
Grade		**<0.001**
First year	22.16 ± 4.41	
Second year	23.29 ± 4.17	
Third grade	**23.82 ± 3.93**	
Fourth year	23.21 ± 4.93	
Fifth grade	21.87 ± 4.27	
Gender^*^		**<0.001**
Male	**23.25 ± 4.44**	
Female	22.95 ± 4.23	

^*^One-way ANOVA.

### 3.5 Plans for a future career

According to our research, 88.45% of medical students plan to pursue a master’s degree after graduation. Among them, the majority (52.97%) expressed a preference for enrolling in a professional master’s program, while 34.91% indicated an interest in pursuing an academic master’s degree. Furthermore, 4.32% of students stated their intention to pursue a master’s degree abroad, and 7.79% remained uncertain about their future academic goals. These results suggest that medical students generally have a positive outlook on continuing their education and recognize the importance of research in shaping their future careers.

Clinical medicine majors exhibited the strongest desire to continue their education, with 91.2% of five-year clinical students intending to pursue a master’s degree. Conversely, nursing majors showed the least inclination to further their education, with only 75.3% expressing such aspirations. This difference may be attributed to the varying future growth needs and job prospects across different disciplines. Among the cohorts, fourth-year undergraduates demonstrated the highest motivation to pursue further education, with 91.3% indicating a desire to earn a master’s degree, and more than half of them opting for specialized master’s programs.

### 3.6 Barriers

The most frequently cited barriers were lack of knowledge (71.0%), limited exposure/opportunities (70.6%), time constraints (56.1%), and inadequate mentorship (51.8%). These barriers were more prominent among students in clinically-oriented majors and in lower academic years. The obstacles to undergraduate research participation varied depending on the major. Students enrolled in five-year clinical medicine, basic medicine, medical technology, and Chinese medicine programs most commonly identified an insufficient knowledge foundation related to research as their main barrier. In contrast, students from other fields most often cited a lack of available research opportunities as their greatest obstacle.

### 3.7 Teachers’ questionnaire

Teachers with a variety of academic backgrounds and teaching experiences participated in this questionnaire, representing different sections. Notably, 47.1% of faculty members were involved in teaching undergraduate major courses, while 66.7% were supervising undergraduate internships. Furthermore, 62.7% served as undergraduate instructors, classroom teachers, or counselors; 17.6% taught graduate major courses; 21.6% held positions as master’s degree instructors; and 25.5% were engaged in teaching within hospital settings.

The majority of faculty members expressed the belief that research is crucial for undergraduate students (4.00 ± 0.849) and emphasized the necessity of incorporating research instruction at the undergraduate level (4.14 ± 0.939). However, respondents indicated that the environment for undergraduate research appeared to be relatively weak (3.86 ± 0.960), and they assessed the quality of undergraduate research exposure as suboptimal (3.49 ± 1.065). Additionally, a significant proportion (72.8%) of graduate advisors regarded prior undergraduate research experience as a priority criterion for graduate school admissions, noting that students with such experience tend to demonstrate greater academic potential and are more likely to complete projects independently.

Unfortunately, many students mentored by these faculty members lack undergraduate research experience, which may explain their advocacy for more undergraduate research opportunities. Most faculty members (79.59%) incorporate research into their teaching processes, with 45.1% involving undergraduate students in research trials and 78.43% mentoring them in innovative training programs. Faculty members who participated in this survey identified basic knowledge of research as the most deficient aspect of undergraduate research education (74.5%), followed by creative thinking skills (52.9%), research abilities (51%), passion for research (27.5%), and education on research integrity (11.8%).

In terms of teaching undergraduate research, faculty members indicated that literature search and screening were the most essential training needs (98%), followed by data organization and statistical analysis (82.4%), dissertation writing skills (82.4%), clinical experiment design (74.5%), and finally, basic laboratory training, project design, and application writing at 64.7 and 51%, respectively. The characteristics of the 51 faculty members who participated in this study are presented in Table 5.

**Table 5:** The teachers’ demographics and academic characteristics.

Gender	
Male	22 (43.1)
Female	29 (56.9)
Top degree	
Bachelor’s	3 (5.9)
Master’s	13 (25.5)
Doctorate	11 (21.6)
Postdoctoral	24 (47.1)
Professional title	
Assistant Professor/Physicia	3 (5.9)
Lecturer/Attending Physician	23 (45.1)
Associate Professor/Associate Physician/Associate Researcher	21 (41.2)
Professor/Chief Physician/Researcher	4 (7.8)

## 4 Discussion

This study presents the first nationwide assessment of research engagement, attitudes, and barriers among undergraduate medical students in China. Our findings indicate that although a substantial proportion of students have participated in at least one research activity, considerable challenges persist—especially regarding access, mentorship, and research literacy.

Compared with global trends, China’s undergraduate medical research participation rate (70.6%) remains lower than that of countries like the United States (83.9%) and several high-income nations reported in Funston et al. (14, 15). While methodological variations exist—such as differences in medical education models, research incentives, and curriculum integration—this disparity underscores the need for systemic reforms in China’s research education landscape. Students from eight-year programs and disciplines such as preventive and basic medicine demonstrated the highest engagement. These findings are consistent with their stronger academic infrastructures and research-oriented curricular components (16). Conversely, five-year clinical students reported lower involvement, likely due to heavier clinical loads and reduced emphasis on research exposure. Our findings echo existing literature (9, 17), which shows that structural differences in curriculum significantly shape research participation.

Undergraduate medical students’ interest and motivation in research activities is a source of motivation that encourages their engagement in research activities along with knowledge of research techniques (18, 19). While most students recognize research as a valuable and integral part of their education, the motivations driving this interest differ across countries. For instance, many students pursue research to enhance their competitiveness for residency placements (20), strengthen their curriculum vitae or résumés, or gain access to U.S. residency programs as international medical graduates (21). In contrast, Chinese medical students are primarily motivated by a genuine interest in research. Unfortunately, this interest has not translated into appropriate research outputs, nor has it inspired clinical undergraduates to pursue careers as physician-scientists (6). One critical challenge in undergraduate research education is the failure to nurture students’ individual research interests (20). Neglecting these interests can hinder their ability to gain a deeper understanding and practical application of medical research. To address this, research education must focus on cultivating students’ curiosity and providing opportunities for hands-on involvement in real research projects.

The identification of barriers—particularly insufficient knowledge, limited opportunities, and lack of guidance—points to multifaceted constraints. These span personal readiness, institutional capability, and socio-cultural expectations (12, 13). Addressing these will require a combination of targeted interventions: embedding research methodology courses early in the curriculum, expanding faculty-led mentoring programs, and enhancing access to structured, hands-on research opportunities.

In undergraduate research education, determining what makes a research teaching model effective remains a significant challenge (22, 23). One potential solution is the establishment of mentor-led research interest groups (23). Additionally, incorporating interactive sessions and workshops covering all aspects of medical research into the early years of the curriculum could be a transformative change for medical undergraduates (24). Medical education instructors should prioritize the development of research skills across all areas of the undergraduate curriculum. To provide students with the fundamental research competencies necessary for their career paths and to build a solid foundation for future research careers, the core curriculum must guarantee that every graduate gains relevant and applicable research expertise. Educators must also acknowledge that students’ training needs, motivation for research, and research proficiency are influenced by factors such as prior educational background, previous research experience, and cultural and gender-related factors (18).

Gender disparities observed in our study—where male students reported higher research involvement and more favorable attitudes—align with international findings (25–27). Although gender parity in academic medicine has improved over the last 20 years, women still lag behind men in terms of rank, retention, leadership, number of articles published, and impact of publications. Social norms, access to mentorship, and implicit biases within academic environments may account for such differences (28). However, it is essential to recognize the role of institutional gender equity policies and cultural factors, which were not specifically examined in this study but warrant future exploration.

Notably, our faculty survey complements student perspectives, reinforcing the finding that knowledge deficits are the most pressing concern. Teachers also emphasized the need for stronger institutional support and recommended prioritizing literature search, data analysis, and scientific writing skills. These insights suggest that aligning curricular objectives with faculty capacity and student needs is essential to cultivating a research-oriented academic culture.

Future studies could explore regional or institutional differences more systematically. Longitudinal follow-up of this cohort may provide insights into how undergraduate research participation influences postgraduate outcomes, such as specialty choice, publication record, or pursuit of academic careers.

## 5 Strengths and limitations

A major strength of this study lies in its nationwide scope, encompassing responses from a diverse array of institutions, programs, and student demographics. The use of validated instruments and a relatively high response rate contribute to the robustness of the findings. Furthermore, the inclusion of faculty perspectives offers a more holistic view of undergraduate research challenges.

However, certain limitations must be acknowledged. First, as a cross-sectional study, causal relationships cannot be established. Second, the reliance on self-reported data may introduce bias, including social desirability and recall bias. Third, while efforts were made to reduce duplication and enhance geographic diversity, the use of convenience sampling may have introduced selection bias. Fourthly, the exclusion of certain contextual variables—such as institutional research funding levels or gender support policies—may limit the interpretation of subgroup differences. Finally, despite IP screening, shared networks or devices may have allowed undetected duplicate responses, though manual review minimized this risk.

## 6 Conclusion

Our findings underscore a strong interest and moderate level of engagement in research among Chinese undergraduate medical students. Compared with global trends, China’s undergraduate medical research participation rate (70.6%) remains lower than that of countries like the United States (83.9%) and several high-income nations. However, major barriers such as insufficient research literacy, limited opportunities, and inadequate mentorship continue to constrain their full participation. Gender, major, and academic year emerged as significant predictors of attitudes and involvement. To foster a robust research culture, academic institutions should adopt multifaceted strategies: integrate research training early in the curriculum, promote equitable mentorship, and invest in institutional infrastructure. Aligning these efforts with national medical education reforms will be essential to cultivating the next generation of physician-scientists in China. Future longitudinal studies are recommended to examine how early research experiences influence postgraduate academic performance, career choice, and research productivity over time.

## Data Availability

The raw data supporting the conclusions of this article will be made available by the authors, without undue reservation.

## Footnotes

^1^ https://www.wjx.cn
