# Supplementary material for: Current status of medical research among undergraduate medical students in China: a nationwide questionnaire survey
Source: Front Med (Lausanne). 2025 Jul 10;12:1593233. doi: 10.3389/fmed.2025.1593233 (PMC12286971; doi:10.3389/fmed.2025.1593233)
Supplement: Supplementary file 1 [file Data_Sheet_1.docx]

Supplementary Material

**Questionnaire for the survey of undergraduate medical students' participation in research**

Question 1 What's your name (can be anonymous)？

Question 2 What is your gender?

Question 3 What college are you attending now?

Question 4 What's your major? (multiple choice)

1.Five-year clinical medicine program

2.Eight-year clinical medicine program

3.Preventive medicine

4.Basic medicine

5.Nursing

6.Stomatology

7.Medical technology specialty

8.Traditional Chinese medicine

Question 5 What's your grade?(multiple choice)

1.First year

2.Second year

3.Third grade

4.Fourth year

5.Fifth grade

6.Sixth grade

7.Seventh grade

8.Eighth grade

Question 6 Please rate each of the following research skills before you enrolled in school（1 indicates no understanding, 2 indicates slight understanding, 3 indicates basic understanding, 4 indicates basic mastery, and 5 indicates proficiency in use）：

1.The ability to innovate

2.The ability to design a subject

3.Knowledge of clinical research

4.Knowledge of basic research

5.Knowledge of research methods and processes

6.Searching and screening of literature

7.The use of a literature manager

8.The ability of data organization and statistical analysis

9.The ability of writing a medical paper

10.The overall research ability

Question 7 Please rate each of the following current research skills（1 indicates no understanding, 2 indicates slight understanding, 3 indicates basic understanding, 4 indicates basic mastery, and 5 indicates proficiency in use）：

1.The ability to innovate

2.The ability to design a subject

3.Knowledge of clinical research

4.Knowledge of basic research

5.Knowledge of research methods and processes

6.Searching and screening of literature

7.The use of a literature manager

8.The ability of data organization and statistical analysis

9.The ability of writing a medical paper

10.The overall research ability

Question 8 What is the average amount of time you spend on research per week？

Question 9 What do you know about scientific research?

Question 10 Have you ever participated in scientific research？

Question 11 What are the forms of research activities you have participated in？

Question 12 Have you ever participated in a college student innovation training program or “Challenge Cup” competition?

Question 13 What is your motivation for participating in research activities？

Question 14 What have you gained from participating in research activities?

Question 15 Do you have the following research experience？

1.I have joined research groups

2.I have written a paper

3.I have published a paper

4.I have applied for a patent

5.I have no experience with appeals

Question 16 Please select the option below that you feel best matches your attitude。

Attitude items 1 2 3 4 5

Doing research is fun

Exposure to research opportunities is high

I would like to have more opportunities to participate in research activities

I think research is important in my future medical career

I am willing to participate in an undergraduate research teaching program

Question 17 Are you considering pursuing a master's degree？

Question 18 Are you considering pursuing a doctoral degree？

Question 19 What grade level do you think undergraduates should start being exposed to research？

Question 20 What grade did you start with research?

Question 21 What do you think are the main barriers to undergraduate students participating in research？

Question 22 If an undergraduate research training program were to be conducted，what would you like to be able to learn ？

Question 23 If an undergraduate research training program were to be conducted, what would you like the format of the program to be?
